# Muscle Satellite Cell Heterogeneity: Does Embryonic Origin Matter?

**DOI:** 10.3389/fcell.2021.750534

**Published:** 2021-10-15

**Authors:** Lara Rodriguez-Outeiriño, Francisco Hernandez-Torres, F. Ramírez-de Acuña, Lidia Matías-Valiente, Cristina Sanchez-Fernandez, Diego Franco, Amelia Eva Aranega

**Affiliations:** ^1^Department of Experimental Biology, Faculty of Experimental Sciences, University of Jaén, Jaén, Spain; ^2^Medina Foundation, Technology Park of Health Sciences, Granada, Spain; ^3^Department of Biochemistry and Molecular Biology III and Immunology, Faculty of Medicine, University of Granada, Granada, Spain

**Keywords:** myogenic precursor cells, embryonic myogenesis, adult myogenesis, satellite cell heterogeneity, muscle regeneration

## Abstract

Muscle regeneration is an important homeostatic process of adult skeletal muscle that recapitulates many aspects of embryonic myogenesis. Satellite cells (SCs) are the main muscle stem cells responsible for skeletal muscle regeneration. SCs reside between the myofiber basal lamina and the sarcolemma of the muscle fiber in a quiescent state. However, in response to physiological stimuli or muscle trauma, activated SCs transiently re-enter the cell cycle to proliferate and subsequently exit the cell cycle to differentiate or self-renew. Recent evidence has stated that SCs display functional heterogeneity linked to regenerative capability with an undifferentiated subgroup that is more prone to self-renewal, as well as committed progenitor cells ready for myogenic differentiation. Several lineage tracing studies suggest that such SC heterogeneity could be associated with different embryonic origins. Although it has been established that SCs are derived from the central dermomyotome, how a small subpopulation of the SCs progeny maintain their stem cell identity while most progress through the myogenic program to construct myofibers is not well understood. In this review, we synthesize the works supporting the different developmental origins of SCs as the genesis of their functional heterogeneity.

## Introduction

Muscle repair and homeostasis are mediated by resident stem cells, also called SCs. Anatomically, SCs are located between the myofiber basal lamina and the sarcolemma of the muscle fiber and functionally are quiescent cells. Quiescent SCs are characterized by the expression of the transcription factor Pax7 but, upon acute injury, pathological conditions or muscle homeostasis, they become activated and give rise to myogenic progenitors that massively proliferate, start to express the myogenic regulatory factors (MRFs) Myf5, Myod1, Myf6 (also known as MRF4), and Myog, differentiate and fuse to form new myofibers and restore the muscle tissue. Classically, SCs have been considered a homogeneous population of muscle stem cells. However, a deeper molecular characterization together with grafting experiments have revealed a certain level of SCs heterogeneity in terms of the expression of specific markers and cellular functionality that determine their regenerative potential. It is well known that SCs and developmental myogenic progenitors share the transcriptional program that drives myogenic differentiation and muscle genesis. Several lineage tracing studies have established that SCs originate from myogenic precursors in the dermomyotome. Nevertheless, behavioral heterogeneity has also been observed in myogenic precursors during development, given the differences in proliferative rate observed in dermomyotome-derived progenitors during embryonic myogenesis and MRF expression patterns in fetal and perinatal stages, despite their functional redundancy (Gros et al., 2005; Kassar-Duchossoy et al., 2005; Picard and Marcelle, 2013). Therefore, developmental myogenesis could provide us with an appropriate scenario to better understand satellite heterogeneity.

In this review, we summarize the latest research evidence on SC functional heterogeneity and examine the principles that sustain myogenic progenitor pool diversity of SCs of somitic origin and the implications for the emergence of SC heterogeneity.

## Satellite Cells in the Context of Adult Myogenesis

Skeletal muscle is a heterogeneous tissue that represents one-third of human body mass with a high capability of regeneration throughout life (Janssen et al., 2000). This ability resides mainly in *bona fide* muscle stem cells, SCs, described by electron microscopy in Mauro (1961). The SC population represents a quiescent cell population between the basal lamina and the myofiber plasma membrane and is characterized by the expression of the paired box transcription factor Pax7 (Seale et al., 2000). Although SCs uniformly express Pax7, the role of this gene in the context of muscle stem cell biology has been largely controversial (Oustanina et al., 2004). Therefore, research by Lepper et al. (2009) that conditionally inactivates Pax7 gene expression in adult SCs in the mouse shows that Pax7 could be dispensable for proper muscle stem cell function and muscle regeneration in adult skeletal muscle and is only required for myogenic function during the perinatal period. In contrast, subsequent studies, carried out by using similar mouse models, have demonstrated that Pax7 plays an essential role in regulating the myogenic potential and function of satellite cells in both neonatal and adult skeletal muscle, challenged the efficiency for Pax7 deletion in Lepper’s study (Günther et al., 2013; von Maltzahn et al., 2013). Nonetheless, full ablation of Pax7 positive cells in adult mice confirmed that Pax7-expressing cells are essential for acute injury-induced muscle regeneration (Lepper et al., 2011; Sambasivan et al., 2011). In addition, more recent studies have highlighted that SCs are the main stem cell source for muscle regeneration (von Maltzahn et al., 2013).

Upon muscle injury, disease, or adult skeletal muscle homeostasis, SCs are activated to achieve appropriate muscle repair. During this process, an embryonic myogenic transcriptional program is recapitulated (Hernandez-Torres et al., 2017). Pax7 is downregulated and the Myogenic Regulatory Factors (MRFs), members of the basic-helix-loop-helix family, are sequentially upregulated to directly activate SCs toward the myogenic differentiation program and eventual fusion to existing myofibers or to form new myofibers (Weintraub et al., 1991). Myf5 is the first MRF that becomes upregulated in SCs, followed by Myod1, which indicates the myogenic commitment to myoblasts (Rudnicki et al., 1992, 1993). The terminal differentiation to myocyte is regulated by the expression of MRF Myog (Venuti et al., 1995). Along with this myogenic specification, reciprocal inhibition between Pax7 and the MRFs’ Myod1 and Myog is required for an accurate differentiation (Olguin et al., 2007).

In the framework of SC activation, it is interesting to note that cell proliferation takes place through symmetric or asymmetric division. In symmetric division, two identical daughter cells (Pax7 +) are formed, maintaining the SC pool in adult skeletal muscle. Meanwhile, asymmetric division gives rise to both committed (Pax7 + /Myf5 + /Myod1 +) and stem cell (Pax7+) progeny. Committed SC Pax7−/Myf5 + /Myod1 + can also undergo symmetrical division to increase the number of myogenic precursors during muscle regeneration (Motohashi and Asakura, 2014; Feige et al., 2018). A correct balance between symmetric and asymmetric SC division is needed to keep the SC pool together and successfully complete muscle repair throughout life. In some muscle disorders such as Duchenne Muscle Dystrophy (DMD), a decreased asymmetric division leads to a lack of myogenic progenitors and, therefore, to inappropriate muscle homeostasis and flawed regeneration (Dumont et al., 2015). A full understanding of the regulation of the different pathways of SC activation and the mechanism underlying muscle stem cell division decisions helps us to better understand SC biology and function in the context of muscle repair and disease.

## Behavioral Heterogeneity in the Satellite Cell Population

The process by which SCs emerge from their quiescent status and enter the myogenic program is well established; however, several studies have highlighted the existence of different SC responses related to their performance during the muscle repair process (Tierney and Sacco, 2016; Cornelison, 2018). In this section, we examine the different behaviors of SCs and identify different SC subpopulations during their activation and myogenic differentiation in the course of regeneration.

**FIGURE 1:**
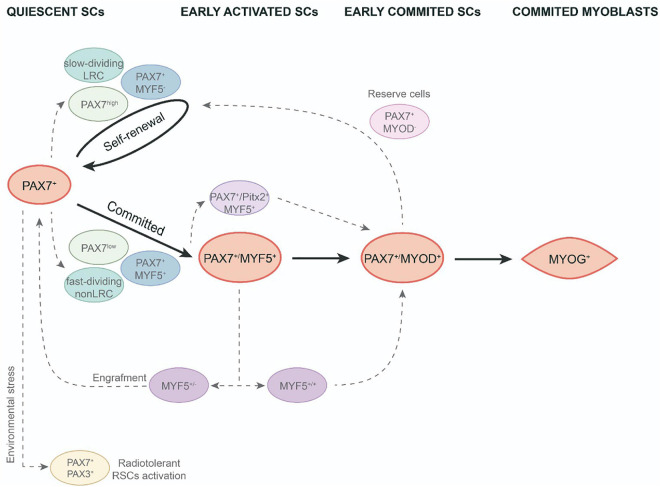
Functional heterogeneity in adult satellite cell population. SCs activation and myogenic differentiation during adult muscle regeneration (black arrows). SC heterogeneity in the course of quiescence, early activation and early commitment of adult SCs (discontinued gray arrows). LRC, label-retaining cells; non-LRC, non-label-retaining cells; RSCs, reserve stem cells; SCs, satellite cells.

Different responses associated with their proliferative capabilities have been identified in the onset of SC activation. An initial study to analyze the cycle time for SCs *in vivo*, revealed that SCs are a mixture of cell populations comprising 80% fast-dividing cells, mainly responsible for providing myonuclei to growing fibers, and 20% slow-dividing cells (Figure 1). The slowest proliferative subpopulation was designed as “reserve cells” as they are likely to undergo asymmetric division and thus produce stem-like cells that maintain the SC pool (Schultz, 1996). According to these observations, a study of *in vitro* myogenic differentiation of fresh isolated SCs performed by Ono et al. (2012) also discriminated a minor subpopulation of activated and undifferentiated SCs from fast-dividing cells that divides more slowly and displays a long-term self-renewal ability, after their passage tended to immediately differentiate without producing any additional proliferative progeny. The undifferentiated very slow-dividing SC subpopulation was characterized by a higher expression of Id1 protein, an inhibitor of myogenic differentiation (Ono et al., 2012). Interestingly, when slow-dividing SCs were transplanted, they also produced regenerating myofibers *in vivo* (Ono et al., 2012). These authors interpreted that, in a regenerative scenario, slow-dividing cells have a preferential effect for engraftment.

As observed above, asymmetric division allows to preserve the pool of SCs for any required future regeneration as one of the two daughter cells maintains its stem cell status. In this context, Shinin et al. (2006) revealed a non-random process of DNA segregation during asymmetric division, in which the cell that retains the old DNA template expresses the quiescent SC marker Pax7. This observation connects with the hypothesis of the immortal DNA strand proposed by Cairns et al., which supports stem cells that segregate the original DNA and reduces accumulated DNA replication errors in the tissue (Cairns, 1975). Thus, non-random DNA segregation of some SCs could give rise to a subpopulation with a robust stem-cell status. Regarding these findings, using transgenic Tg:Pax7-nGFP mice, Rocheteau et al. (2012) showed that quiescent SCs display different levels of Pax7 expression linked to different stemness properties. Thus, the Pax7^high^ subpopulation is more prone to asymmetric DNA segregation, retaining the old DNA strand, and exhibiting a low metabolic status as dormant adult SCs (Rocheteau et al., 2012). Nevertheless, this dormant state is reversible as, after several transplantations, the Pax7^high^ subpopulation was able to give rise to both Pax7^high^ and Pax7^low^ SCs and thus allow proper muscle repair (Rocheteau et al., 2012; Figure 1).

To study these different SC proliferative dynamics, Chakkalakal et al. (2014) used a TetO-H2B-GFP reporter. They found that approx. 30% of SCs retained the H2B-GFP label (LRCs), whereas the vast majority lost it (non-LRCs). Both cell populations are formed at birth and during the juvenile period they set up as different populations with divergent behaviors that will persist throughout adult life. LRCs were less differentiated and, when transplanted, showed similar properties to stem cells, generating self-renewal and differentiated cells. Meanwhile, non-LRCs only undergo myogenic differentiation (Figure 1). They found that the CIP/KIP family members p21cip1 (Cdkn1a) and p27kip1 (Cdkn1b) were responsible for maintaining these LRCs at the stemness stage (Chakkalakal et al., 2014). Together, these findings indicate the existence of a less common subpopulation of SCs with a greater stem-cell status. These cells are preferably in a dormant status, less prone to proliferate but, once activated, they undergo asymmetric division with non-random DNA strand segregation. So, after cell transplantation, they can produce both progenies, the stem cells and the myogenic committed cells. Consequently, it has been postulated that this subpopulation could be the true muscle stem cells responsible for maintaining SC populations long-term throughout life.

To elucidate the mechanism underlying the diverse pattern of activations in different SC populations, using an Myf5^nlacZ/+^ mouse line, Zammit et al. identified the main SC subpopulation expressing both the stem cell marker CD34 and also the Myf5 transcription factor, thus revealing that quiescent SCs are committed to myogenesis. Yet, a minor subpopulation CD34-/Myf5- maintains the committed-lineage population (Beauchamp et al., 2000). To investigate the impact of Myf5 expression on different SC populations, Gayraud-Morel et al. (2012) studied the transcriptome and functional behavior of SC heterozygous for Myf5 in mice. They showed that Myf5 ± SCs were transcriptionally committed to myogenic differentiation, as demonstrated by the higher expression levels for some differentiation genes such as Myod1, Myog, and/or contractile protein genes. Interestingly, this heterozygous Myf5 SC population displays a high Tie2 expression, a marker for SC self-renewal. In addition, when they analyzed the functional behavior of this SC population, they found that Myf5 ± similarly contributes to muscle regeneration after an injury compared to wild type. Moreover, Myf5 ± cells more efficiently replenish the SC niche and, after a second injury, were capable of contributing to new myofiber formation (Gayraud-Morel et al., 2012; Figure 1). These findings highlight the relevance of Myf5 expression levels in functional heterogeneity in early committed SCs.

Satellite cell heterogeneous behavior has been also observed during the early *in vitro* differentiation process of activated SCs. *In vitro* experiments in the C2C12 cell line and freshly isolated myofibers from EDL found an SC subpopulation of reserve-like cells in activated progenitors that also expressed MRF Myod1 (Yoshida et al., 1998; Zammit et al., 2004). When kept in culture, Pax7 + /Myod1 + cells mostly downregulate Pax7 and differentiate into myotubes, however, a minor subset of this cell population downregulated Myod1 and remained in cell culture as Pax7 + /Myod1- reserve cells (Figure 1). This subpopulation can re-enter the cell cycle, upregulate Myod1 and give rise to either myogenic committed cells (Myod1 + /Myog +) or new reserve cells (Pax7 + /Myod1-) (Yoshida et al., 1998; Zammit et al., 2004). These research studies highlight the fact that a subpopulation of committed progenitors can revert to quiescence to maintain self-renewal of the muscle stem cell pool. Besides, it has been shown that satellite cell–derived myoblasts isolated from adult mice lacking the *MyoD* gene (*MyoD^–/–^*) exhibit a high resistance to apoptosis through microRNA (miRNA)-mediated down-regulation of Pax3; as well as a significantly higher engraftment rate after intramuscular transplantation (Asakura et al., 2007; Hirai et al., 2010). In addition, a subset of SCs in the adult muscle also express the transcription factor Pitx2 and *in vitro* gain and loss of function experiments indicate that Pitx2 has an effect expanding SC-derived myogenic committed population (Vallejo et al., 2018; Figure 1). Finally, it is interesting to note that proximity to the blood vessels it has been associated with SC self-renewal (Verma et al., 2018).

In terms of the gene expression profile, intriguingly, another subset of SCs also expresses Pax3 (a paralog of Pax7) in some muscles (Relaix et al., 2006). Importantly, it has recently been stated that Pax3 + /Pax7 + SCs can play specific roles under environmental stimulus. Hence, Pax7 + /Pax3 + SC subpopulations demonstrate different behaviors when submitted to radiation stress or environmental pollutants (der Vartanian et al., 2019; Scaramozza et al., 2019). By utilizing Mx1-Cre transgenic reporter mice, which allow to trace resident stem cells in most adult tissues, Scaramozza et al. (2019) showed that freshly isolated Mx1 + SCs and Mx1 − SCs exhibited similar Pax7 expression levels but only Mx1 + SCs were enriched for Pax3. The Mx1 + /Pax7 + /Pax3 + SCs displayed reduced levels of reactive oxygen species (ROS) in both basal conditions and after irradiation. Due to their relatively low abundance and consistent with reserve stem cell (RSC) characteristics, these cells possess important stem cell activity upon transplantation but only make a slight contribution to muscle repair. Conversely, an extensive clonal expansion of Mx1 + /Pax7 + /Pax3 + SCs allows extensive muscle repair as well as niche repopulations upon selective pressure of radiation stress. However, the lack of Pax3 in these cells increased ROS components and diminished cell survival and stress tolerance. These findings show that a discrete subpopulation of radiotolerant RSCs of SCs undergo clonal expansion under severe stress (Scaramozza et al., 2019; Figure 1). In the same line, der Vartanian et al. (2019) have shown a bimodal response to environmental stress for SC subpopulations. Therefore, the exposure to a ubiquitous and highly toxic pollutant (2,3,7,8-tetrachlorodibenzo-p-dioxin; TCDD) leads to a specific loss of PAX3- MuSCs in adult skeletal muscle, whereas PAX3 + MuSCs are preserved. Nevertheless, PAX3-positive MuSCs become sensitized to environmental stress when the PAX3 function is lost and PAX3-mediated induction of mTORC1 is required for protection (der Vartanian et al., 2019). All these data highlight a functional heterogeneity of SCs related to their response to environmental stress controlled by PAX3.

It is interesting to note that, since SC functional heterogeneity in adult muscles is substantively based on the variability of their cell proliferation and the expression of different myogenic specification and/or determination genes, developmental myogenesis could offer the potential to understand this diversification behavior in the SC compartment.

## Embryonic Myogenesis Overview: Progenitor and Myoblast Populations

### Primary and Secondary Myogenesis

In vertebrates, prenatal skeletal muscle development takes place through two rounds of myogenesis. Between embryonic day E9.5 and E14.5 in mice, a primary round (also termed embryonic myogenesis) takes place to give rise to primary muscle fibers. This is followed by a secondary round of myogenesis (also termed fetal myogenesis), which gives rise to the majority of skeletal-muscle fibers present at birth (Biressi et al., 2007a; Tajbakhsh, 2009; Deries and Thorsteinsdóttir, 2016). Each round of myogenesis requires the proliferation, determination, and commitment of progenitors to myoblasts, differentiation of myocytes, and fusion of myocytes toward multinucleate myofibers.

**FIGURE 2:**
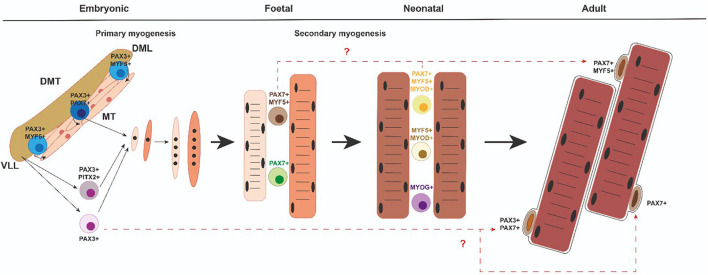
Myogenic progenitors and satellite cells. Diverse myogenic cell populations emerging in the course of developmental and neonatal myogenesis could give rise to different subsets of SC within adult muscle. DMT, dermomyotome; MT, myotome; DML dorsomedial lip of the dermomyotome; VLL, ventrolateral lip of the dermomyotome.

All muscles in the trunk and limbs derive from myogenic precursor cells (MPCs), which are present in somites, transient structures that form pairwise on either side of the neural tube. The somites are initially a spherical accumulation of cells but they soon subdivide into two compartments, the ventral mesenchymal sclerotome (ST) and the dorsal epithelial dermomyotome (DMT) (Hernandez-Torres et al., 2017, 2018; Figure 2). ST contains precursor cells that will give rise to the axial skeleton and ribs (Hernandez-Torres et al., 2017, 2018). DMT encloses proliferating progenitors of all skeletal muscles of the trunk, brown fat, endothelial cells, and dorsal dermis. MPCs from the dorsomedial (DML), ventrolateral (VLL) (Figure 2), rostral (ROL), and caudal lips (CAL) of the epithelial DMT undergo an epithelial–mesenchymal transition (EMT) and accumulate underneath, where they differentiate to form the myotome (MT) (Figure 2), the first muscle fibers of the embryo (Buckingham and Relaix, 2015; Deries and Thorsteinsdóttir, 2016; Hernandez-Torres et al., 2017, 2018). Later, MPCs from the central portion of the remaining DMT also reach the MT and thus contribute to its growth. The epaxial MT (located in the dorsomedial position) gives rise to the deep back muscles and the hypaxial DMT (located in the ventrolateral position) will form the body wall muscles, the diaphragm, and the intercostal muscles (Buckingham and Relaix, 2015; Deries and Thorsteinsdóttir, 2016; Hernandez-Torres et al., 2017, 2018). At the limb level, MPCs from the hypaxial DMT undergo an EMT and migrate toward the fore and hind limbs to form dorsal and ventral muscle masses in the limb bud mesenchyme (Biressi et al., 2007a; Deries and Thorsteinsdóttir, 2016; Hernandez-Torres et al., 2017, 2018). After MPCs reach their anatomical goals, they begin to differentiate to form primary muscle fibers that express a specific set of proteins such as the slow MyHC and myosin light chain 1 (MyLC1, Myl1) (Kelly et al., 1997).

During a later fetal or secondary phase (E14.5–17.5 in mouse) characterized by growth and maturation of each muscle anlagen, a group of remaining MPCs either fuse with each other or with the primary fibers and give rise to secondary or fetal fibers that express specific markers such as β-enolase, Nfix, or MyLC3 (Myl3) (Keller et al., 1992; Kelly et al., 1997; Fougerousse et al., 2001; Messina et al., 2010). At this time, the fibers also start to express fast MyHC isoforms (van Horn and Crow, 1989). Throughout secondary myogenesis, muscle growth is performed essentially by cell fusion and the addition of myonuclei from proliferating progenitors (White et al., 2010). During this phase, a subset of MPCs will also form the pool of adult muscle stem cells—the SCs (Gros et al., 2005; Kassar-Duchossoy et al., 2005; Relaix et al., 2005). Therefore, secondary myofibers form the majority of muscle and this process is complete after birth in mice (Relaix et al., 2005). Importantly, after birth (0–21 days postnatally), neonatal myogenesis is necessary for proper muscle growth and myofiber growth takes place with the rapid increase of myonuclear cell numbers, while some neonatal progenitors reach a unique anatomical position and locate between the plasmalemma and basement membrane of the adult myofibers. Thus, they are called SCs (Mauro, 1961).

In the gene regulatory cascade that regulates embryonic myogenesis, Pax3 and Pax7 genes are indispensable upstream regulators for specification and migration of MPCs in the trunk (Buckingham and Relaix, 2007; Lagha et al., 2009; Buckingham and Rigby, 2014; Mayeuf-Louchart et al., 2014, 2016). Pax3 is already expressed in presomitic mesoderm, maintaining its expression in the epithelial somite (Tajbakhsh and Cossu, 1997). With further development, Pax3 expression is progressively restricted to the dermomyotome and later to the DML and VLL of the DMT. Pax3 is also expressed in the hypaxial DMT-derived cells during E10.5–E13.5. Pax7 expression begins later and is restricted to the central portion of the DMT. In the limbs, Pax7 expression begins at E11.5 in hypaxial DMT-derived cells. Pax7 expression is maintained during fetal and neonatal myogenesis as well as in adult SCs (Figure 2). Pax3 is not generally expressed in muscle after E13.5. However, a few adult SCs also are Pax3 + (Relaix et al., 2006; Figure 2). It is of note that Pitx2 transcription factor is also present in some of the myogenic cell progenitors in the VLL of the DMT as well as in migrating muscle precursor cells toward the limb buds (Hernandez-Torres et al., 2017). From a functional point of view, Pax3 is necessary for somite segmentation and formation of the dermomyotome lips (Schubert et al., 2001; Relaix et al., 2004). Although primary myotome is present in Pax3 mutant mice, limb muscles are absent, mainly due to defective pre-myoblast cell migration (Relaix et al., 2004; Messina and Cossu, 2009). However, Pax7 mutant mice show no defects in embryonic or fetal myogenesis but Pax7 is required to maintain adult SCs and make them function (Seale et al., 2000; Oustanina et al., 2004; Kuang et al., 2006; Relaix et al., 2006; von Maltzahn et al., 2013). In addition, Pax7 is sufficient to lead to myogenic specification *in vitro* (Seale et al., 2004). Interestingly, Pax3/Pax7 double mutant mice do not give rise to any limb muscle but neither does the primary myotome initially form (Relaix et al., 2005). Overall, all these data indicate that the gradual expression of Pax3 or Pax7 is critical for initiating the generation and survival of embryonic, fetal, neonatal, and adult muscle progenitors.

Myogenic determination and differentiation of all MPCs are controlled by the myogenic regulatory factor (MRF) family: Myf5, Myod1, Myf6, and Myog (Buckingham and Rigby, 2014). Myf5, Myod1, and Myf6 have traditionally been classified as determining factors that regulate cell fate and lineage progression from MPCs to myoblasts. Myog later acts to regulate myoblast terminal differentiation, myofiber maturation, and size (Zammit, 2017). Thus, the MRFs are expressed in myoblasts, myocytes, and myofibers at different stages of myogenesis (Buckingham and Rigby, 2014; Comai and Tajbakhsh, 2014).

During somite maturation, Myf5 expression is activated in the epaxial and hypaxial domains of the DM. Shortly after, the Myf5 expressing cells move beneath the DM to form the primary myotome (Relaix and Marcelle, 2009; Nitzan and Kalcheim, 2013). Myod1 is also present in these DM domains (Ott et al., 1991; Tajbakhsh et al., 1997; Venters et al., 1999; Cinnamon et al., 2001; Kablar et al., 2003; Kassar-Duchossoy et al., 2004; Buckingham and Rigby, 2014). At the same time, Myf6 expression is initiated in the DM and MT but is downregulated at E12.5 in mice, suggesting that Myf6 could have a function in MPC determination (Summerbell et al., 2002; Kassar-Duchossoy et al., 2004). Curiously, around fetal day E14.5 in mice, Myf6 transcription is reactivated in all skeletal muscles (Bober et al., 1991; Hinterberger et al., 1991; Summerbell et al., 2002). This first wave of Pax3 + MPCs that progressively express Myf5, Myod1, and Myf6 rapidly gives rise to DESMIN + myoblasts that form the MT (Kassar-Duchossoy et al., 2005; Comai and Tajbakhsh, 2014; Figure 2). Soon after, a second wave of Pax3/Pax7-expressing MPCs is displaced from the central DM toward MT, activates Myf5 and Myod1, and differentiates (Relaix et al., 2005; Buckingham and Relaix, 2007; Figure 2). A subset of this second wave of MPCs does not activate Myf5 and Myod1 but retains a proliferative status, thus forming a reserve of MPCs for later embryonic and fetal development (Relaix et al., 2005; Buckingham and Relaix, 2007). It is interesting to highlight that the Pax3 positive MPCs from the hypaxial DM that exit their epithelial structure, begin a long-range migration to the limbs, retain their proliferative status and avoid MRF expression (Vasyutina and Birchmeier, 2006; Figure 2). These migrating MPCs begin to express Myod1 and Myog after reaching the limbs buds, form ventral and dorsal cell masses, and start to differentiate into myoblast, myocytes, and muscle fibers (Lee et al., 2013).

### Different Progenitors and Myoblast Populations

As a result of the multiple rounds of skeletal myogenesis, embryonic and fetal myoblasts emerge as different cell populations. It has been shown that these distinct groups of myoblasts display distinctive *in vitro* characteristics such as different morphologies, culture media conditions, and drug responses and give rise to myofibers with distinct morphologies. Therefore, embryonic myoblasts are elongated cells in culture and differentiate into mononucleated or oligonucleated myotubes, with the highest tendency to differentiate and give rise to smaller colonies when cultured *in vitro*, while fetal myoblasts exhibit a more triangular shape (Biressi et al., 2007a). Moreover, embryonic myoblast differentiation is not affected by molecules such as TGFβ, BMP-4, or phorbol esters and present a different sensitivity to merocyanine 540 (Nameroff and Rhodes, 1989). However, fetal myoblasts proliferate to a limited extent in response to growth factors, differentiate into large multinucleated myotubes, and their differentiation is inhibited by TGFβ, BMP-4, and phorbol esters. Moreover, genetic analyses of Pax3/7 and Myf5/Myod1/Mrf4 transcription factors and genome-wide expression analysis have revealed that embryonic and fetal myoblasts are specified by different transcription factor cocktails and express different genes (Kassar-Duchossoy et al., 2004, 2005; Relaix et al., 2006; Biressi et al., 2007b).

An intriguing issue in the field is whether embryonic, fetal, and adult myoblasts derive from common or different progenitor populations. In this sense, genetic labeling and ablation of myogenic progenitors have revealed that Pax3 + and Pax7 + cells contribute differentially to embryonic and fetal limb myogenesis. Consequently, Pax3 + /Pax7 + cells contribute to muscle and endothelium, are required for embryonic myogenesis, and generate Pax7 + cells. Later, Pax7 + cells produce fetal myogenesis (Hutcheson et al., 2009; Figure 2). Moreover, these two embryonic and fetal limb myogenic populations have autonomous genetic requirements. In the somite, Beta-Catenin is necessary for proper dermomyotome and myotome formation and limb progenitor delamination. However, Beta-catenin is not required for embryonic myoblast specification or differentiation in limbs but is important for determining fetal progenitor number and myofiber number and type (Hutcheson et al., 2009). On the other hand, from E5 to E17 in chicks and E12 to E15 in mice, different myogenic progenitors have been identified, depending on their proliferative capabilities, as a minor Pax7 + slow-cycling less-differentiated population and a major Pax7 + /Myf5 + highly proliferative pool of cells further engaged in the myogenic program (Picard and Marcelle, 2013; Figure 2). These results add levels of complexity to cellular heterogeneity during vertebrate skeletal muscle development. Besides, another intriguing topic in the field is whether myoblast diversity is due to different intrinsic changes or whether changes in myogenic cells occur as a consequence of the extrinsic environment (Murphy and Kardon, 2011).

## Does the Embryonic Origin of Satellite Cells Underlie Functional Heterogeneity?

Although it is well accepted that SCs arise from Pax3/Pax7-expressing DMT-cells (Gros et al., 2005; Relaix et al., 2005; Schienda et al., 2006; Hutcheson et al., 2009; Lepper and Fan, 2010) the embryonic origin of SCs is still an open issue. In this sense, a seminal lineage tracing study using a Myf5-Cre-stop-flox-YFP reporter mouse line demonstrated the existence of a small subpopulation of ∼10% of SCs that had never previously expressed Myf5 (Kuang et al., 2007). These Myf5-YFP-reporter-negative cells were more prone to divide in an asymmetrical apical–basal manner, generating a more committed YFP-reporter-positive cell and a negative cell that self-renewed. Moreover, this subset of Myf5-YFP-reporter-negative cells had a much higher capacity to repopulate the SC niche (compared to Myf5-YFP-reporter-positive SCs) when transplanted into PAX7-null animals, indicating that these cells could retain more robust self-renewal capabilities. Conversely, non-*Myf5* expression SCs were more prone to myogenic differentiation in *in vivo* engraftment assays (Kuang et al., 2007; Figure 1). However, other Cre/lox lineage analyses revealed that essentially all adult SCs associated with limb and body wall musculature and the diaphragm and extraocular muscles originate from Myod1 + progenitors (Kanisicak et al., 2009). These data have highlighted some still unsolved questions about the embryonic origin of SCs. For example, can SC progenitors also constitute a heterogeneous cell pool? In other words, can SCs originate from different dermomyotome/embryonic and/or fetal progenitors that give rise to functionally-diverse populations of SCs?

It is very well accepted that SCs derive from the same embryonic “founder” cells as the muscle in which they reside. In this context, it is interesting to highlight that during the multiple growth phases during myogenesis, SCs arise from stem and progenitor cells that resist differentiation throughout life and eventually arrive at the SC compartment. To further distinguish myogenic progenitors that form the muscle prenatally from the juvenile and adult SC population, different rounds of founder stem cells have been termed FSC1, FSC2, and FSC3 (Tajbakhsh, 2009). FSC1 has been defined as cells that express Pax3 and form the myotome; FSC2 as progenitors released from the central dermomyotome that express Pax3/Pax7, and FSC3 as progenitors that migrate from the ventral dermomyotome to form the skeletal muscles in the limbs, diaphragm, and tongue. FSC3 cells also express Pax3 but start to express Myf5/Myod1/(Mrf4) once they reach their destination (Tajbakhsh, 2009). In this regard, it has been proposed that FCS1 is exhausted early in the embryo but FSC2 and FSC3 remain and contribute to the majority of SCs (Tajbakhsh, 2009). On the other hand, juvenile SCs, located beneath the basement membrane, emerge from about 2 days before birth in mice but continue to proliferate until about 12 days postnatally. Then, at around 2 weeks, quiescent “adult” SCs can definitely be identified (Tajbakhsh, 2009). It has been assumed in the field that the founder stem cells that establish the muscles before birth give rise to juvenile SCs at postnatal stages that encourage muscle growth and regeneration (Sambasivan and Tajbakhsh, 2007). However, mononuclear “juvenile SCs” are heterogeneous as they include: (1) Pax7 + /(Myf5 + /Myod1 +) identified as future SCs; (2) myoblasts that upregulate or downregulate Pax7 but express Myf5 and Myod1 and can give rise to future SCs or differentiate, and (3) differentiated Pax7-/Myog + (Kassar-Duchossoy et al., 2005; Sambasivan and Tajbakhsh, 2007; Figure 2). All these “juvenile” cells must be distinguished from those in G0 and emerge from approx. 2–3 weeks after birth as adult quiescent SCs (Shinin et al., 2006). In this scenario, several questions need to be addressed: How can “satellite” cells be differentiated from fetal and/or juvenile myoblast precursors? Could the heterogeneous cell populations emerging at the fetal and/or juvenile stages give rise to functionally different populations with different quiescent properties and functions? Future works in this area could help to advance the characterization of the embryonic origin of “different” adult SC populations.

At this point, it is interesting to recall that approx. 90% of quiescent SCs also express Myf5 despite Pax7 expression being present in all SCs in the adult muscle. Further analysis of the developmental stage at which the precursors of SCs first express muscle determination genes using the Tamoxifen-inducible Myf5^CreER^ mouse line revealed that a significant number of SCs develop from cells that expressed Myf5 for the first time at the fetal stage (∼E15) in the mouse (Biressi et al., 2013). Nevertheless, it has also been taken into account that Pax3 is present in some SCs in several skeletal muscles (Relaix et al., 2006) and that they retain long-term self-renewal and proliferation as well as a better response to stress and/or environmental injuries (Yang et al., 2016; der Vartanian et al., 2019; Scaramozza et al., 2019). These data generate new questions about links between SC functional heterogeneity in adults and their embryonic/fetal origin. For example, do Pax7 + /Myf5 + and Pax7 + /Pax3 + SC populations in adult muscle share common fetal myogenic progenitors that eventually downregulate Pax3 and upregulate MRFs in some of their daughter cells? Alternatively, do Pax7 + /Myf5 + SC populations originate from fetal progenitors while Pax3 + /Pax7 + SCs derive from Pax3 + embryonic progenitors that remain in fetal, juvenile, and adult muscle as Myf5 negative cells? An additional question arises as to whether different cell populations emerge due to intrinsic changes or extrinsic environmental factors possibly regulating such differences. A deeper analysis of the molecular signals that differentially regulate the different phases of myogenesis will help us to better address these questions.

## Discussion/Concluding Remarks

Muscle regeneration is mediated by SCs that lie in close proximity to the muscle fibers. Stem cell heterogeneity is recognized as functionally relevant for tissue homeostasis and repair. Muscle SCs are a heterogeneous population regarding cell cycle progression, lineage commitment, ability to self-renew and repopulate the niche, and response to environmental stress. To sustain proper muscle regeneration, quiescent SCs activate, proliferate, and recapitulate the embryonic myogenic program to differentiate and form new myoblasts that give rise to muscle fibers and/or fuse with pre-existing fibers. In this scenario, it is interesting to note that these different SC behaviors are mainly supported by diverse cell subpopulations with different myogenic determination gene expression profiles and proliferative capabilities. Although it is very well accepted that SCs originate from dermomyotome progenitors, the developmental origin of muscle stem cells has not been fully clarified. As several previous studies have indicated that diverse myogenic precursors emerge that also display differences in proliferation rates and myogenic gene expression during embryonic, fetal, and neonatal myogenesis, developmental myogenesis could be an excellent platform to better understand SC behavior in adult muscles. In this regard, further analysis of the molecular mechanisms underlying the emergence of different myoblast populations during the different rounds of embryonic, fetal, and neonatal myogenesis could enable us to decipher whether diverse muscle stem cell populations could arise from those diverse cell populations. Finally, another critical point that needs to be addressed is how a subset of myogenic progenitors leave the cell cycle, resist myogenic differentiation throughout development, and give rise to quiescent SCs.

## Author Contributions

LR-O and AA conceived the structure and content. FH-T, LR-O, and AA designed and produced the figures. FH-T, FR, LM-V, CS-F, and DF revised the manuscript critically for important intellectual content. AA corrected, edited, and approved the final version of the document to be published. All authors contributed to the article and approved the submitted version.

## Conflict of Interest

The authors declare that the research was conducted in the absence of any commercial or financial relationships that could be construed as a potential conflict of interest.

## Publisher’s Note

All claims expressed in this article are solely those of the authors and do not necessarily represent those of their affiliated organizations, or those of the publisher, the editors and the reviewers. Any product that may be evaluated in this article, or claim that may be made by its manufacturer, is not guaranteed or endorsed by the publisher.
